# Differential Degradation of Full-length and Cleaved Ataxin-7 Fragments in a Novel Stable Inducible SCA7 Model

**DOI:** 10.1007/s12031-012-9722-8

**Published:** 2012-02-25

**Authors:** Xin Yu, Abiodun Ajayi, Narasimha Rao Boga, Anna-Lena Ström

**Affiliations:** Department of Neurochemistry, Stockholm University, 106 91 Stockholm, Sweden

**Keywords:** Aggregation, Ataxin-7, Autophagy, Polyglutamine, Proteasome, SCA7

## Abstract

Spinocerebellar ataxia type 7 (SCA7) is one of nine neurodegenerative disorders caused by expanded polyglutamine repeats, and a common toxic gain-of-function mechanism has been proposed. Proteolytic cleavage of several polyglutamine proteins has been identified and suggested to modulate the polyglutamine toxicity. In this study, we show that full-length and cleaved fragments of the SCA7 disease protein ataxin-7 (ATXN7) are differentially degraded. We found that the ubiquitin–proteosome system (UPS) was essential for the degradation of full-length endogenous ATXN7 or transgenic full-length ATXN7 with a normal or expanded glutamine repeat in both HEK 293T and stable PC12 cells. However, a similar contribution by UPS and autophagy was found for the degradation of proteolytically cleaved ATXN7 fragments. Furthermore, in our novel stable inducible PC12 model, induction of mutant ATXN7 expression resulted in toxicity and this toxicity was worsened by inhibition of either UPS or autophagy. In contrast, pharmacological activation of autophagy could ameliorate the ATXN7-induced toxicity. Based on our findings, we propose that both UPS and autophagy are important for the reduction of mutant ataxin-7-induced toxicity, and enhancing ATXN7 clearance through autophagy could be used as a potential therapeutic strategy in SCA7.

**Electronic supplementary material:**

The online version of this article (doi:10.1007/s12031-012-9722-8) contains supplementary material, which is available to authorized users.

## Introduction

Spinocerebellar ataxia type 7 (SCA7) is an autosomal dominant neurodegenerative disorder characterized by neuronal death in the retina, cerebellum, and brainstem (Harding and Deufel 1993). The disease is caused by expansion of an unstable CAG repeat in the 5′-end of the SCA7 gene resulting in an expanded polyglutamine domain in the ataxin-7 (ATXN7) protein (David et al. 1997). ATXN7 is a widely expressed protein (Jonasson et al. 2002; Lindenberg et al. 2000) and a subunit of the STAGA (SPT3-TAF(II)31-GCN5L acetylase) complex (Helmlinger et al. 2004; Palhan et al. 2005). STAGA regulates transcription through its histone acetylation and de-ubiquitination activity (Helmlinger et al. 2004; McCullough and Grant 2010; Palhan et al. 2005). However, the exact role of ATXN7 in STAGA and how this function is affected by the polyglutamine expansion are still unclear (for review, see McCullough and Grant 2010).

Besides SCA7, eight other neurodegenerative disorders including Huntington’s disease (HD), dentatorubral pallidolusian atrophy, spinal bulbar muscular atrophy, and SCA1-3, 6, and 17, caused by CAG/glutamine expansions, have been identified (Katsuno et al. 2008). These disorders are commonly known as polyglutamine (polyQ) diseases and are characterized by aggregation of the expanded polyQ protein into nuclear and/or cytoplasmic inclusions in the neurons of patients. Several studies have suggested that proteins containing expanded polyQ domains misfold and assume an abnormal β-sheet conformation which results in their aggregation (for review, see Hands and Wyttenbach 2010). A correlation between the ability of the polyglutamine protein to aggregate and cause toxicity has been shown; however, whether misfolded monomers, oligomers, or large inclusions are the major toxic species is still unclear (Davies and Scherzinger 1997; Hands and Wyttenbach 2010; Scherzinger et al. 1999).

Protein degradation systems are crucial for clearance of damaged and misfolded proteins which could cause cellular toxicity. The ubiquitin–proteasome system (UPS) and autophagy are the two main protein degradation pathways in mammalian cells (for review, see Wong and Cuervo 2010b). UPS mainly degrades short-lived proteins in a two-step process: (1) the enzyme catalyzed attachment of multiple ubiquitin molecules to the target protein, which signals its targeting to cytoplasmic or nuclear proteasomes, and (2) degradation of the ubiquitinated target protein by the 26S proteasome (Wong and Cuervo 2010b). Mutant polyglutamine proteins have been reported to be resistant to UPS clearance and even disrupt UPS activity (Bence et al. 2001; Bennett et al. 2007; Holmberg et al. 2004; Jana et al. 2001; Verhoef et al. 2002). However, other studies have questioned these findings (Iwata et al. 2009; Maynard et al. 2009; Michalik and Van Broeckhoven 2004; Pratt and Rechsteiner 2008). In contrast to UPS, autophagy only occurs in the cytoplasm and can degrade organelles and cytoplasmic material as well as damaged proteins. Three types of autophagy have been identified: macroautophagy, microautophagy, and chaperone-mediated autophagy (Wong and Cuervo 2010b). Macrophagy is the major form of autophagy and begins with the engulfment of cytosolic material by a double membrane resulting in structures called autophagosomes. The autophagosomes then fuse with lysosomes leading to the degradation of the engulfed material by lysosomal hydrolases (Wong and Cuervo 2010b). Degradation of some expanded polyQ proteins via macroautophagy has been observed (Qin et al. 2006; Ravikumar et al. 2002; Young et al. 2009), and enhancing this pathway was shown to ameliorate toxicity in some, but not all polyQ disease models (Menzies et al. 2010; Nisoli et al. 2010; Ravikumar et al. 2004; Tanaka et al. 2004).

In this study, we show that both UPS and autophagy degradation are important to reduce ATXN7 toxicity. However, the two pathways are used differently to degrade full-length and cleaved ATXN7 fragments. While full-length mutant ATXN7 as well as endogenous ATXN7 was primarily found in the nucleus and degraded by UPS in both PC12 and HEK 293T cells, cleaved ATXN7 fragments were detected in both the nucleus and the cytoplasm and degraded to a similar level by autophagy and UPS. Furthermore, pharmacological activation of autophagy ameliorated the toxic effect of mutant ATXN7 in a novel stable inducible PC12 cell model. The work presented here suggests that both UPS and autophagy are important for the clearance of mutant ATXN7, and enhancing autophagic activity could potentially be used as a therapeutic strategy in SCA7.

## Materials and Methods

### Plasmids

Plasmids FLQ10 and FLQ65, encoding N-terminal Flag and C-terminal myc-tagged full-length ATXN7 referred to as ATXN7Q10-Myc and ATXN7Q65-Myc, have been previously reported (Strom et al. 2005). To generate plasmids FLQ10-pTRE-tight and FLQ65-pTRE-tight encoding N-terminal FLAG-tagged and C-terminally GFP-tagged full-length ATXN7 with 10 (ATXN7Q10-GFP) or 65 (ATXN7Q65-GFP) glutamine, respectively, FLAG-ATXN7 was excised from FLQ10 and FLQ65 using *Sac*I and *Sal*I and ligated into pEGFP-N1 in frame with the C-terminal GFP tag. FLAG-ATXN7-GFP was then excised from pEGFP-N1 in two pieces (*Nhe*I–*Sal*I and *Sal*I–*Not*I) and ligated into the pTRE-tight vector (Clontech) digested with *Nhe*I and *Not*I.

### Cell Culture, Transfections, and Generation of Inducible PC12 Cell Lines

Human Embryonic Kidney 293T (HEK 293T) cells were maintained in Dulbecco’s modified Eagle’s medium (DMEM, Invitrogen) supplemented with 10% fetal bovine serum (FBS, Invitrogen) and 1% penicillin/streptomycin (Invitrogen) at 37°C, 5% CO_2_. For transient transfections, 7 × 10^5^ HEK 293T cells were seeded in six-well plates and transfected 24 h later using polyethyleneimine (CELLnTEC) according to the product protocol.

Stable PC12 cell lines were generated by co-transfecting the Tet-off PC12 cell line (Clontech) with the FLQ10-pTRE-tight or FLQ65-pTRE-tight construct and a linear hygromycin marker (Clontech). Individual hygromycin-resistant colonies were isolated using 200 μg/ml hygromycin and 200 μg/ml G418. Doxycycline was used at 1 μg/ml to keep ATXN7-GFP expression off during the selection process. Screening for clones showing correct inducible ATXN7 expression was done by removing doxycycline from the media and western blot of cell extracts. Acquired PC12 stable cell lines were grown at 37°C and 5% CO_2_, in DMEM (Invitrogen) supplemented with 10% horse serum (Invitrogen), 5% tet system approved fetal bovine serum (PAA), 100 μg/ml G418 (Invitrogen), 100 U/ml penicillin G sodium, 100 μg/ml streptomycin sulfate (Invitrogen), 100 μg/ml hygromycin (Invitrogen), and 1 μg/ml doxycycline (Sigma) when desired.

### Treatments

Autophagy and UPS inhibitors or inducers were used at the following final concentrations: 200 ng/ml rapamycin (R0395, Sigma), 10 mM NH_4_Cl (A0171, Sigma), 200 nM epoxomycin (E3652, Sigma), 10 mM 3-MA (M9281, Sigma), and 10 mM trehalose (Sc-202368, Santa Cruz). Cells were treated for 24 or 48 h as indicated in the figures. The effect of epoxomycin on UPS was verified by detection of poly-ubiquitinated proteins. The inhibition or stimulation of autophagy following treatments was confirmed by analysis of the microtubule-associated protein 1 light chain 3 (LC3) protein, which when macroautophagy is activated associates to autophagosomes in its LC3 II cleaved and lipidated form (Mizushima et al. 2008, 2010; Rubinsztein et al. 2009). Inhibition of autophagy by 3-MA results in decreased LC3 II levels, while inhibition of autophagy by NH_4_Cl results in increased levels due to an arrest in the degradation of autophagosomes. Induction of autophagy by rapamycin results in increased LC3 II levels.

### Cell Lysis, Western Blot, and Calculation of ATXN7 Half-lives

HEK 293T or PC12 cells were harvested with radioimmunoprecipitation assay (RIPA) buffer (Millipore) supplemented with protease inhibitor cocktail (Sigma) and phenylmethylsulfonyl fluoride (PMSF, Sigma). The supernatant which we call the soluble fraction was collected after centrifugation at 21,000 × *g* at 4°C for 10 min. Protein concentrations were determined with Bradford assay (Bio-Rad) and 10–20 μg of extract was subjected to SDS-PAGE. Proteins were transferred onto nitrocellulose membrane (Whatman), blocked in 10% milk-TBST (100 mM Tris-buffered saline pH 7.4, 0.1% tween-20), and incubated with primary antibodies in 1% milk-TBST. Membranes were then washed three times with TBST, incubated with secondary antibody in 1% milk-TBST, and again washed three times with TBST. The protein of interest was visualized using SuperSignal West Pico chemiluminescent substrate or SuperSignal West extended duration substrate kits (Pierce) followed by film exposure or detection by a ChemiDoc XRS+ imaging system (Bio-Rad). Primary antibodies were used at the following concentrations: ATXN7 (SCA7 1-135) (Strom et al. 2005) 1:700, actin (SC-1616, Santa Cruz) 1:500, ubiquitin (SC-8017 Santa Cruz) 1:500, LC3 (PM036, MBL International) 1:1,000, tubulin (T9026, Sigma-Aldrich) 1:1,000, and histone H3 (ab1791, Abcam). Signal intensities of target bands were quantified by Image lab software (Bio-Rad). The relative intensity of the target protein in the control and treated samples was acquired by first normalizing the target band with the corresponding actin intensity. The normalized intensity in the control or treated samples was then divided by the sum of the normalized intensities of the target protein in the control and all treated samples. The quota for the control sample was set to 100% and all treated samples in that experiment were shown as percent compared to control.

The half-lives, i.e., the time required to reduce the ataxin-7 content by 50%, were calculated from three separate experiments using the formula: ${M_t} = {M_0} \times {\left( {{1}/{2}} \right)^t}^{{/T}}$, where *T* = the half-life, *M*_0_ = the amount at 0 h, and *M*_*t*_ = the amount at 12 h for soluble ataxin-7 and 288 h for aggregated ATXN7 material.

### Filter Trap Assay

**Fig. 1:**
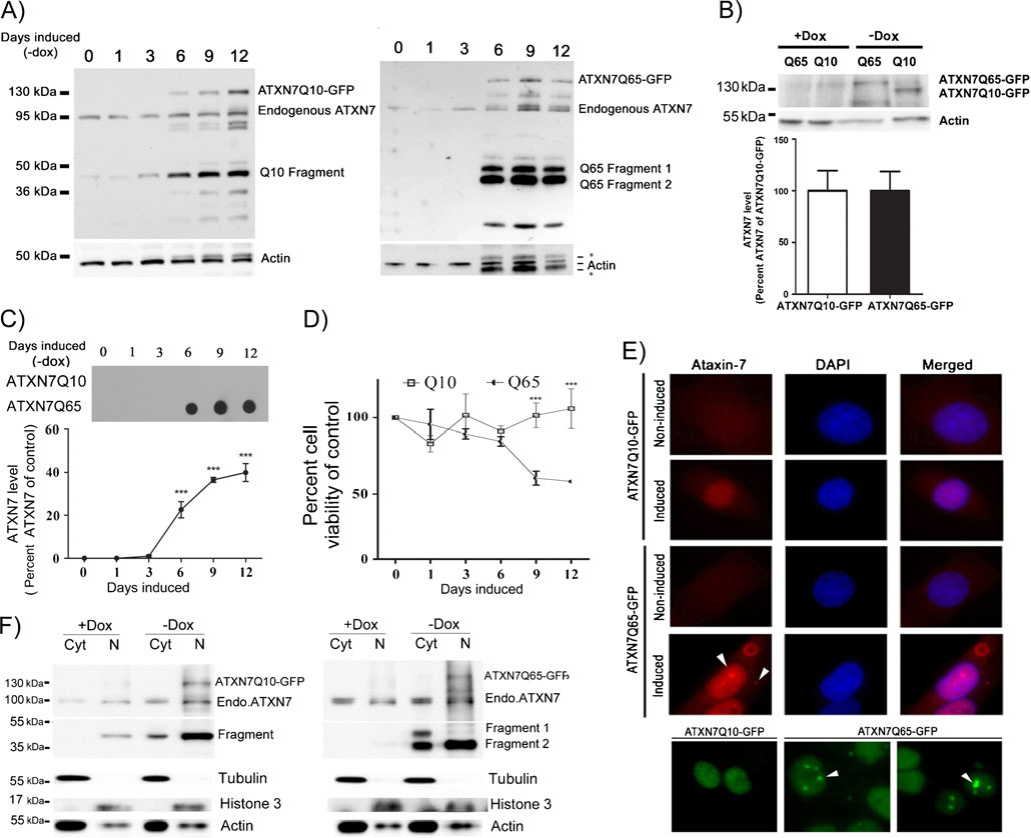
Mutant ATXN7 aggregates and causes toxicity in a stable inducible PC12 cell model. **a** Expression of GFP-tagged ATXN7 with 10 (ATXN7Q10-GFP, *left panel*) or 65 (ATXN7Q65-GFP, *right panel*) glutamines after induction (doxycycline removal) of FLQ10 and FLQ65 stable PC12 cell lines for the indicated number of days. Western blot analysis using an ATXN7 antibody detected endogenous ATXN7, the respective full-length transgenic proteins and cleaved ATXN7 fragments. Actin was used as loading control. *Asterisk* indicates background from previous probing. **b** The expression level of ATXN7Q10-GFP and ATXN7Q65-GFP is not statistically different in FLQ10 and FLQ65 cells 12 days after induction. Actin was used for normalization and quantifications were done from three independent experiments. **c** Analysis of ATXN7 aggregation. Cells induced to express ATXN7Q10-GFP or ATXN7Q65-GFP for the indicated numbers of days were harvested and RIPA insoluble fractions were subjected to filter trap assay. **d** Viability of FLQ10 and FLQ65 cells induced to express ATXN7Q10-GFP or ATXN7Q65-GFP for the indicated number of days. Viability was measured by WST-1 assay and normalized against the protein content. **e** Microscopic analysis of ATXN7 in induced and non-induced FLQ10 and FLQ65 cells using an ATXN7 antibody (*upper panel*) and GFP fluorescence (*lower panel*). *Arrows* indicate inclusions. **f** Cytoplasmic (*Cyt*) and nuclear (*N*) proteins were separated by fractionation and the localization of ATXN7 analyzed by western blot. For quantifications, all data are shown as means ± SEM from three independent experiments. ***p < 0.001

Cells were lysed using RIPA buffer and centrifuged at 21,000 × *g* as described above. After centrifugation, the supernatant, i.e., the soluble fraction, was removed and the pellet containing RIPA insoluble material was washed two times with 1× PBS and resuspended in 50 μl DNaseI reaction buffer containing 4 U of DNaseI enzyme (EN0521, Fermentas). The resuspended pellet, called the insoluble fraction, was incubated at 37°C for 1 h, and the Bradford assay (Bio-Rad) was then used to determine the protein concentration in the sample. SDS and dithiothreitol (DTT) were then added to a final concentration of 2% and 100 mM, respectively, before the sample was heated at 95°C for 5 min. Insoluble fractions were loaded and vacuum-filtered through a 0.2-μm pore size nitrocellulose (Whatman) membrane using a Bio-Rad dot-blot apparatus. A 0.1% SDS solution was added to each dot-blot slots twice for washing and the membrane was then removed from the dot-blot and blocked in 10% milk-TBST. The membrane was then subjected to immunoblotting using ATXN7 antibody as described above. Following immunoblotting, signal intensities of ATXN7 dots were quantified by Image Lab software (Bio-Rad) and normalized against the protein concentrations. For analysis of aggregation during induction (Fig. 1c), the average intensity from each dot was divided by the sum of the intensities of ATXN7 from all time points in that experiment. The results are shown as percent aggregation of the total aggregation at a certain time point. In all other experiments where the aggregation in non-treated and treated cells was compared, the intensity from the untreated control sample was set to 100%, and all treated samples in that experiment were shown as percent compared to control.

### Cellular Fractionations

FLQ10 or FLQ65 cells, non-induced or induced to express ATXN7 for 9 days, were resuspended and washed two times with PBS. After centrifugation for 5 min at 1,850 × *g*, the PBS was removed and the packed cell volume (PCV) was estimated. The cells were resuspended in 5xPCV volume of hypertonic lysis buffer (10 mM HEPES pH 7.9, 1.5 mM MgCl_2_, 10 mM KCL, 1 mM EDTA, freshly supplemented with 0.5 mM DTT, protease inhibitor cocktail (Sigma), and 0.2 mM PMSF (Sigma)) and allowed to swell on ice for 15 min. Cells were collected again by centrifugation as above and resuspended in 2xPCV volume of hypertonic buffer, and the plasma membrane was lysed using a 27-gauge needle. After centrifugation at 11,000 × g at 4°C for 20 min, the supernatant, i.e., the cytoplasmic fraction, was collected. To obtain the nuclear proteins, the pellet was further resuspended in a high salt buffer (20 mM HEPES pH 7.9, 1.5 mM MgCl_2_, 400 mM NaCl, 20% glycerol, 2 mM EDTA, freshly supplemented with the same inhibitors as above) for 30 min to lyse the nuclei. After centrifugation at 20,000 × g for 15 min at 4°C, the supernatant, i.e., the nuclear fraction, was collected. Protein concentrations were determined using the Bradford method and fractions were analyzed using western blot as described above.

### WST-1 Cell Viability Test

FLQ10 and FLQ65 cells, non-induced or induced for 0, 2, 5, 8, or 11 days, were seeded into 96-well plates at 2 × 10^4^ cells/well. The following day, WST-1 assay was performed according to the manufacturer’s protocol (630118, Clontech). For evaluation of the effect of different treatments on viability, non-induced or FLQ65 cells induced for 5 days were seeded at 2 × 10^4^ cells/well. The following day, UPS or autophagy inhibitors/activators were applied as described above for 24 or 48 h before the WST-1 assay was performed. Absorbance (450–690 nm) was measured on a Digiscan absorbance reader (Labvision). After the WST-1 assay, the protein concentration in each well was determined using the Lowry assay (Bio-Rad) and cell viability was normalized against the protein concentration. The value obtained from the untreated non-induced sample was set to 100%.

### Microscopy and Inclusion Counting

Non-induced PC12 cells or PC12 cells with ATXN7 expression induced for 5 days were seeded onto gelatin-coated cover slips. For analysis of GFP fluorescence, cells were allowed to grow for 72 h before they were washed with PBS, fixed for 30 min in 4% paraformaldehyde in 0.1 M PBS, and analyzed by microscopy. For ATXN7 staining and inclusions counting, the indicated inhibitor was added 24 h after seeding and the cells analyzed after an additional 48 h. For analysis, cover slips were washed and fixed as described above, before they were blocked in 10% heat-inactivated FBS in 0.1 M PBS with 0.1% Triton X-100 (PBST) for 30 min. Cover slips were then incubated with ATXN7 primary antibodies (Strom et al. 2005) diluted 1:500 in 2% FBS-PBST overnight at room temperature (RT). Following primary antibody incubation, sections were washed with PBST and incubated with 4′,6-diamidino-2-phenylindole dihydrochoride (Sigma) at 1:7,500 and Alexa fluor 594 anti-rabbit (Molecular Probes) at 1:300 in 10% FBS-PBST at RT for 1 h. Cover slips were then washed with PBST and mounted using a Vectashield-mounting medium. Fluorescence microscopy was carried out using a Leica DM IRBE epifluorescence microscope with a ×100 objective. To quantify the number of cells with inclusions, the total number of cells and the number of cells with ATXN7-positive inclusions were counted in 10 randomly chosen view fields (approximately 200–300 cells) for each treatment in three independent experiments.

### Statistical Analysis

Statistical analysis of western blots, filter trap, and inclusion counting data were done by one-way ANOVA followed by Tukey’s post hoc test using GraphPad Prism 5.0.

## Results

### Expression of Expanded ATXN7 in a Novel Inducible PC12 Cell Line Leads to Aggregation and Toxicity

In order to compare the degradation pathways for wild-type and mutant ATXN7 and to study the effect of autophagy activation on mutant ataxin-7-induced toxicity, we constructed stable inducible PC12 cell lines expressing N-terminal FLAG- and C-terminal GFP-tagged human ATXN7 with 10 or 65 glutamines using the Tet-off expression system. Using this system, the expression of the corresponding proteins named ATXN7Q10-GFP and ATXN7Q65-GFP is induced upon removal of doxycline from the media. One ATXN7Q10-GFP clone (FLQ10 line) and one ATXN7Q65-GFP (FLQ65 line) which had similar induction patterns (Fig. 1a) and expression levels (Fig. 1b) of the respective transgenic proteins after induction were chosen for further studies. Clear expression of the transgenic ATXN7 was detectable from day 6 onwards in both these cell lines (Fig. 1a). Whereas no ATXN7 aggregation was detected in ATXN7Q10-GFP cells, analysis of ATXN7Q65-GFP cells revealed the presence of aggregated ATXN7 material from day 3 onwards using the filter trap method (Fig. 1c). Viability assays showed that induction of ATXN7Q65-GFP caused a progressive reduction in viability, and 12 days after induction, a 41.3% decrease in viability was observed (Fig. 1d). No statistical significant change in viability was detected in ATXN7Q10-GFP expressing cells on any day after induction (Fig. 1d).

Besides visualizing the full-length transgenic proteins, the antibody SCA7 1-135 (Jonasson et al. 2002; Strom et al. 2002) directed against the ATXN7 N-terminal also detected endogenous ATXN7, as well as several ATXN7 fragments in western blot experiments (Fig. 1a). In ATXN7Q10-GFP expressing cells, high levels of one Q10 fragment between 40 and 50 kDa were observed, whereas two major fragments around 45 kDa (Q65 fragment 1) and 40 kDa (Q65 fragment 2) were detected in ATXN7Q65-GFP cells. Truncated N-terminal ATXN7 fragments with similar sizes have been reported in many other SCA7 models, and caspase-7 cleavage of ATXN7 at amino acids 266 and 344 has been reported (Young et al. 2007).

Microscopic analysis revealed low levels of ATXN7 in non-induced PC12 cell lines; however, after induction, a stronger predominantly nuclear expression of ATXN7 was detected in both ATXN7Q10-GFP and ATXN7Q65-GFP expressing cells (Fig. 1e). The nuclear localization was confirmed by cell fractionations, which revealed the presence of both full-length as well as Q10 and Q65 fragments in the nuclei after induction (Fig. 1f). However, the fragments could also be detected in the cytoplasm. In fact, the larger Q65 fragment 1 was predominantly detected in the cytoplasm (Fig. 1f). Microscopic analysis of inclusion formation revealed both nuclear and cytoplasmic ATXN7-positive inclusions in induced FLQ65 cells, with 74.6 ± 4.3% of ATXN7Q65-GFP expressing cells containing inclusions 8 days after induction (Fig. 1e).

### Expansion of the Polyglutamine Domain Increases the Stability of ATXN7 Through Aggregation

**Fig. 2:**
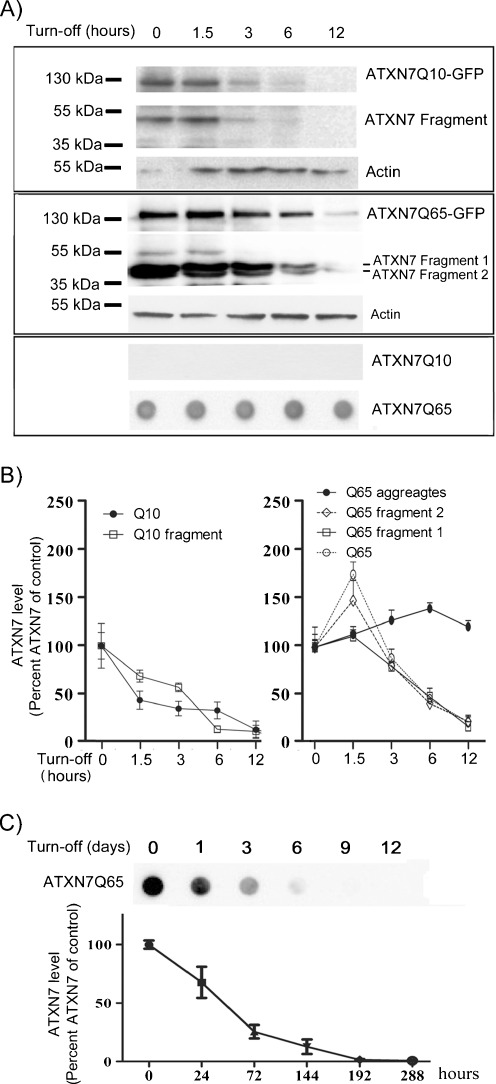
Expansion of the polyglutamine domain stabilizes the ATXN7 protein. **a** Clearance of soluble full-length ATXN7, soluble ATXN7 fragments, and aggregated ATXN7 material. Doxycycline was added (turn off) to the media of FLQ10 or FLQ65 cells induced to express ATX7Q10-GFP or ATXN7Q65-GFP for 10 days and the ATXN7 clearance analyzed during 12 h. *Upper panel*: ATXN7 western blot analysis of ATXN7Q10-GFP cell extracts, *middle panel*: western blot for ATXN7Q65-GFP cells, and *lower panel*: filter trap analysis for ATXN7Q65-GFP cells. **b** Quantification of the clearance from three independent experiments. The ataxin-7 intensity was normalized with actin (loading control) and the average value of 0 h from three independent experiments was set to 100%. **c** Analysis of clearance of aggregated ATXN7Q65 material during 288 h using the filter trap assay. *Upper panel*: a representative blot, *lower panel*: quantification of clearance based on three independent experiments. All data are shown as means ± SEM. *p < 0.05

The ability to switch the ATXN7 expression on or off in our stable PC12 cell models allowed us to compare the stability and clearance rate of ATXN7 carrying a normal or expanded repeat without the use of protein synthesis inhibitors like cycloheximide. This is important since this type of drugs can interfere with protein degradation (Abeliovich et al. 2000; Lawrence and Brown 1993; Ravikumar et al. 2002). To compare the stability of ATXN7Q10-GFP and ATXN7Q65-GFP, the ATXN7 level was analyzed at various time points after doxycycline was added to the media of FLQ10 or FLQ65 cells to turn off further ATXN7 expression. After doxycycline addition, full-length ATXN7Q10-GFP was progressively cleared from the cells with a half-life of approximately 4.5 h (Fig. 2a, b). For the Q10 fragment, a half-life of 4.7 h was observed. In contrast, the level of soluble full-length ATXN7Q65-GFP increased during the first 1.5 h after doxycycline addition after which the level rapidly decreased (Fig. 2a, b). A similar pattern was found for the two main ATXN7 fragments. The initial increase in soluble mutant ATXN7 after doxycycline addition suggests that the mutant protein is more resistant towards degradation and accumulates. This has also been observed in other SCA7 models (Yvert et al. 2001). The level of soluble full-length ATXN7Q65-GFP is determined not only by degradation but also by proteolytic cleavage and aggregation of the full-length protein. We suspected that the rapid clearance of soluble forms of ATXN7Q65-GFP starting from 1.5 h after doxycycline addition could most likely be due to aggregation rather than degradation. Indeed, analysis of the level of aggregated ATXN7 material revealed a progressive increase as the levels of soluble forms of ATXN7Q65-GFP decreased (Fig. 2a, b). Analysis of the stability of the aggregated ATXN7 material revealed a half-life for clearance of approximately 34.3 h (Fig. 2c). These results show that although both Q10 and Q65 ATXN7 are cleared away, expansion of the polyglutamine domain enhances the stability of ATXN7 mainly through rapid aggregation.

### ATXN7 Carrying a Normal 10Q Repeat Is Primarily Degraded by the UPS

**Fig. 3:**
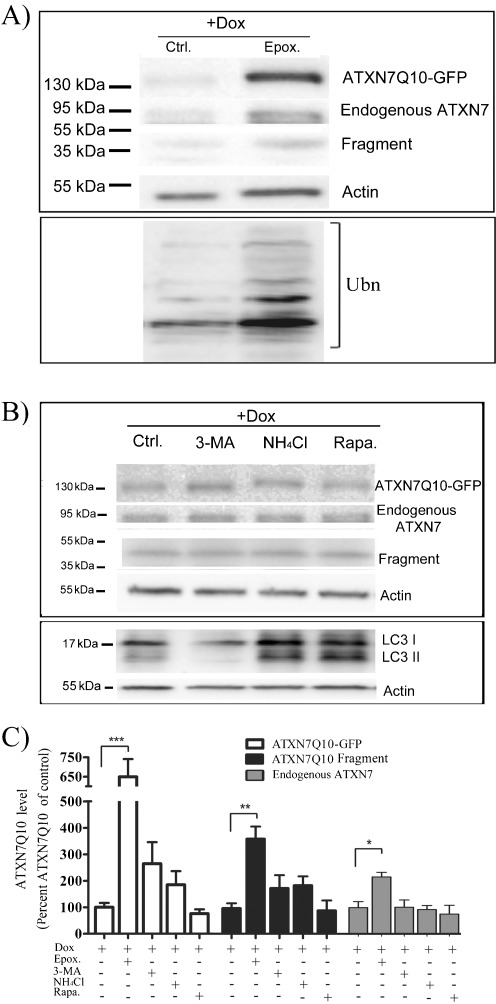
Endogenous ATXN7 and ATXN7Q10-GFP are mainly degraded by UPS in PC12 cells. Expression of ATXN7Q10-GFP was turned off (+Dox) in FLQ10 cells induced for 10 days and the level of endogenous and ATXN7Q10-GFP during 24 h was analyzed in the absence or presence of UPS inhibition, autophagy inhibition, or autophagy activation. **a** Representative western blot analysis of soluble full-length ATXN7Q10-GFP, endogenous ATXN7, and Q10 fragment after inhibition of UPS with 200 nM epoxomycin. Probing for poly-ubiquitinated proteins was done to verify UPS inhibition. **b** Representative western blot analysis of soluble full-length ATXN7Q10-GFP, endogenous ATXN7, and Q10 fragment after autophagy inhibition with 100 mM 3-MA or 10 mM NH_4_Cl, as well as autophagy activation with 200 nM rapamycin. Probing for LC3 II was done to verify the activation or inhibition of autophagy. **c** Quantification of ATXN7 levels from three independent western blots as shown in **a** and **b**. The ATXN7 intensity was normalized with actin (loading control) and the average value of the −Dox control from three independent experiments was set to 100%. Data are shown as means ± SEM. *p < 0.05; **p < 0.01; ***p < 0.001

**Fig. 4:**
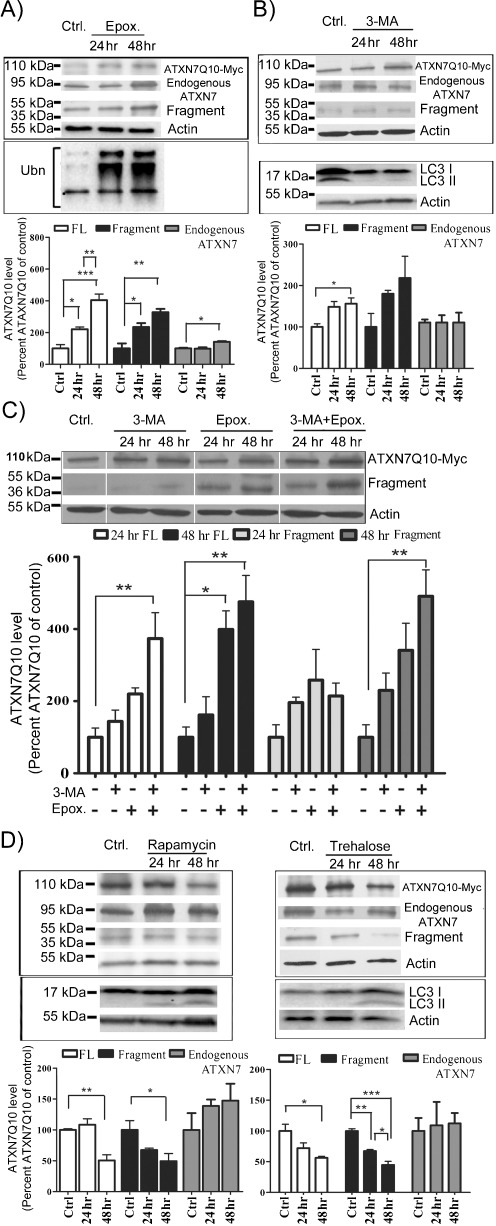
Autophagy and UPS synergistically regulate transient transfected ATXN7Q10 degradation in HEK 293 T cells. The level of endogenous ATXN7 or ATXN7Q10-Myc in transfected HEK 293 T cells was analyzed after a 24- or 48-h incubation with UPS inhibition and/or autophagy inhibition or activation. Non-treated transfected cells were used as control. **a** Effect of UPS inhibition. *Top panel*: a representative western blot for full-length (*FL*) ATXN7Q10-Myc, endogenous ATXN7, and the 40-kDa Q10 fragment. *Middle panel*: UPS inhibition was confirmed by probing for poly-ubiquitinated proteins. *Bottom panel*: Quantification of ATXN7 after normalization with actin (loading control) from three independent experiments with non-treated control cells set to 100%. **b** Effect of autophagy inhibition with 3-MA. *Top panel*: a representative ATXN7 western blot. *Middle panel*: LC3 probing to verify the effect of 3-MA on autophagy. *Bottom panel*: quantification of ATXN7 after normalization with actin (loading control) from three independent experiments with non-treated control cells set to 100%. **c** The effects of combined inhibition of UPS and autophagy on full-length (*FL*) ATXN7Q10-Myc and the Q10 fragment. Representative western blot (*top panel*) and quantification from three independent experiments (*bottom panel*). For quantifications, ATXN7 was normalized with actin (loading control) and non-treated control cells set to 100%. **d** Effect of activation of autophagy with rapamycin or trehalose on endogenous ATXN7, full-length ATXN7Q10-GFP, and Q10 ATXN7 fragment. *Top panels*: representative western blots, *bottom panels*: quantifications from three independent experiments. For quantifications, ATXN7 was normalized with actin (loading control) and non-treated control cells set to 100%. LC3 probing was done to verify the effect of the treatment on autophagy (*middle panels*). For all graphs, data are shown as means ± SEM. *p < 0.05; **p < 0.01; ***p < 0.001

UPS and autophagy are the two main protein degradation pathways in mammalian cells. To determine whether these pathways play a role in the clearance of ATXN7 carrying a normal repeat, the effect of UPS and autophagy inhibition on endogenous and transgenic ATXN7Q10 levels was analyzed (Figs. 3 and 4). We used epoxomycin as a selective proteasome inhibitor (Garcia-Echeverria 2002; Meng et al. 1999) as other commonly used inhibitors such as MG132, ALLN, or lactacystin also have been reported to interfere with autophagic pathways (Abeliovich et al. 2000; Lawrence and Brown 1993; Ravikumar et al. 2002). Treating PC12 or HEK 293T cells with epoxomycin led to a statistical accumulation of endogenous ATXN7 in both cell types (Figs. 3a and 4a). Similarly, application of the inhibitor to FLQ10 PC12 cells or transfected HEK 293T cells decreased the clearance of transgenic ATXN7Q10. In FLQ10 PC12 cells, a sixfold accumulation of full-length ATXN7Q10-GFP could be observed after co-treatment with epoxomycin and doxycycline compared to control cells treated with only doxycycline (Fig. 3c). A fourfold increase in the major Q10 fragment was also observed (Fig. 3a, c). In HEK 293T cells transfected to express myc-tagged ATXN7Q10, treatment with epoxomycin for up to 48 h resulted in a circa fourfold accumulation of full-length ATXN7Q10-Myc and an approximately threefold increase of the Q10 fragment (Fig. 4a). In all experiments, the inhibition of UPS was confirmed by ubiquitin western blot showing accumulation of poly-ubiquitinated proteins after epoxomycin treatment (Figs. 3 and 4). Taken together, these data suggest that UPS is important for the clearance of endogenous as well as transgenic ATXN7Q10 in both PC12 and HEK 293T cells.

Inhibition of autophagy by either 3-MA, an inhibitor of macroautophagy initiation (Cuervo et al. 2004; Qin et al. 2003; Seglen and Gordon 1982), or ammonium chloride (NH_4_Cl), an inhibitor of lysosomal proteolysis (Cuervo et al. 2004), had no effect on the level of endogenous ATXN7 in either PC12 or HEK 293T cells (Figs. 3b, c and 4b). Similarly, activation of autophagy by rapamycin (Blommaart et al. 1995; Ravikumar et al. 2004; Sarkar et al. 2009) or trehalose (Sarkar et al. 2007) had no effect on the level of endogenous ATXN7 in either cell line (Figs. 3b, c and 4d; data not shown). These data suggest that autophagy plays a little role in the degradation of ATXN7 with a normal repeat. In agreement with this, no significant decrease in clearance of transgenic ATXN7Q10-GFP or the Q10 fragment could be observed after autophagy inhibition in the stable PC12 cells (Fig. 3b). However, a small but still statistically significant 1.5-fold accumulation of full-length ATXN7Q10-Myc could be observed in transiently transfected HEK 293T cells after autophagy inhibition (Fig. 4b and data not shown). In HEK 293T cells, combined treatment with 3-MA and epoxomycin for 48 h also had an additive effect on the accumulation of full-length ATXN7Q10-Myc and the Q10 fragment (Fig. 4c). Furthermore, stimulation of autophagy decreased both the ATXN7Q10-Myc and Q10 fragment levels (Fig. 4d). The inhibition or stimulation of autophagy following treatments was confirmed by analysis of the microtubule-associated protein 1 LC3 protein; see “Materials and Methods” for more information (Mizushima et al. 2008, 2010; Rubinsztein et al. 2009). These data suggest that autophagy plays a minor role in the degradation of endogenous ATXN7 in both HEK 293T and PC12 cells, as well as degradation of transgenic ATXN7Q10-GFP in PC12 cells. The observed contribution of autophagy to the degradation of ATXN7Q10-Myc in HEK 293T cells could be a result of the high over-expression acquired after transient transfection or indicate a higher usage of autophagy in HEK 293T cells.

### Both UPS and Autophagy Influence the Level of Aggregated Expanded ATXN7

**Fig. 5:**
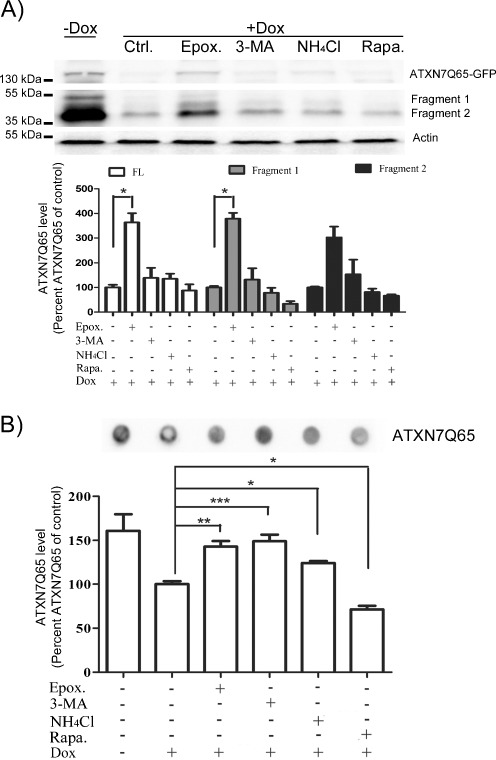
Clearance of soluble ATXN7Q65 by UPS but contribution by autophagy to the reduction of aggregated ATXN7Q65 material in PC12 cells. Expression of ATXN7Q65-GFP was turned off (+Dox) in FLQ65 cells induced for 10 days, and the clearance of ATXN7 during 24 h was analyzed in the absence or presence of UPS inhibition or autophagy inhibition or activation. **a** Analysis of soluble full-length ATXN7Q65-GFP and Q65 fragments 1 and 2. *Top panel*: a representative western blot, *lower panel*: quantification of three independent experiments. For quantifications, ATXN7 was normalized against actin (loading control). **b** Analysis of aggregated ATXN7 material using filter trap assay after UPS and autophagy inhibition or activation. *Top panels*: representative blot, *lower panel*: quantification of three independent experiments with the level in untreated + Dox sample set to 100%. Data are shown as means ± SEM. *p < 0.05; **p < 0.01; ***p < 0001

**Fig. 6:**
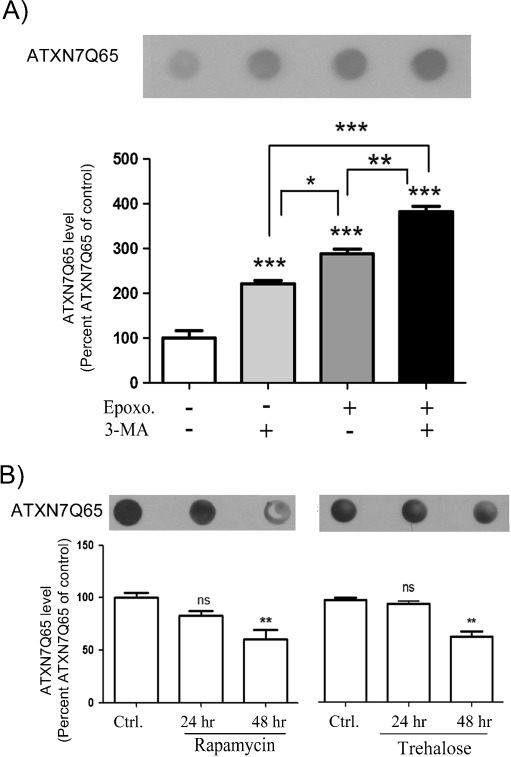
Both UPS and autophagy degrade mutant ATXN7 in HEK 293T cells. HEK 293T cells transfected to express ATXN7Q65-Myc was treated with UPS and or autophagy inhibitors or activators for 24 or 48 h and the level of aggregated ATXN7 material analyzed. Non-treated transfected cells were used as control. **a** Effect of UPS inhibition with epoxomycin, autophagy inhibition with 3-MA, and combined treatment with epoxomycin plus 3-MA. *Top panel*: representative blot, *bottom panel*: quantification. **b** Analysis of aggregated ATXN7 material after activation of autophagy by rapamycin (*left side*) or trehalose (*right side*). For all graphs, quantifications were done from three independent experiments and data are shown as means ± SEM. *p < 0.05; **p < 0.01; ***p < 0.001; *ns* not significant

We next focused on the degradation mechanism(s) of expanded ATXN7. Using the stable FLQ65 PC12 cells, we could observe that UPS inhibition decreased the clearance of both soluble full-length ATXN7Q65-GFP and Q65 fragments after additional ATXN7 expression had been turned off (Fig. 5). An approximate three- to fourfold increase of full-length as well as both major fragments could be detected in epoxomycin-treated cells compared to control cells treated with only doxycycline (Fig. 5a). An approximate 32% increase in aggregated ATXN7 material was also observed after UPS inhibition in the PC12 cells (Fig. 5b). Supporting these data, we could also observe an increase in soluble and aggregated ATXN7 in transfected HEK 293T cells expressing ATXN7Q65-Myc after treatment with epoxomycin (Fig. 6a and data not shown). Since the aggregated material could not be degraded by UPS (Verhoef et al. 2002), these data suggest that UPS degrades soluble full-length ATXN7Q65-GFP or cleaved fragments and the increase in these forms of ATXN7 after inhibition results in increased aggregation.

No significant increase in soluble full-length or ATXN7 fragments was observed after inhibition of autophagy in PC12 cells (Fig. 5a). However, inhibition of autophagy by 3-MA or NH_4_Cl did result in increased levels of aggregated ATXN7 material to a similar degree as UPS inhibition (Fig. 5b). The increase in aggregated material after autophagy inhibition was confirmed in transfected HEK 293T cells expressing ATXN7Q65-Myc (Fig. 6a). In these cells, a clear additive effect could also be observed upon combined inhibition of UPS and autophagy (Fig. 6a). Furthermore, activation of autophagy by either rapamycin or trehalose increased the clearance of aggregated ATXN7 (Fig. 6b). These data suggest that either cleaved ATXN7Q65-GFP fragments and/or aggregated ATXN7 material can be cleared away by autophagy.

### Autophagy Is not Up-regulated in Stable ATXN7Q65-GFP Expressing PC12 Cells

**Fig. 7:**
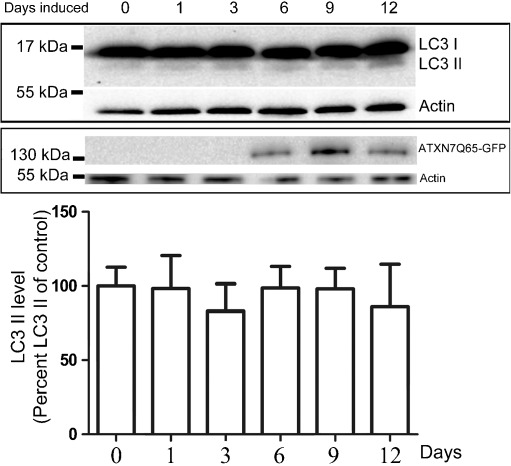
Induction of ATXN7Q65-GFP in PC12 cells does not result in increased autophagic activity. FLQ65 cells were induced to express ATXN7Q65-GFP for the indicated number of days before the cells were harvested and the levels of LC3 I and II analyzed by western blot. Induction of ATXN7Q65-GFP was verified by ATXN7 probing and actin was used as loading control. *Lower panel* shows quantification of the LC3 II level normalized against actin from three independent experiments. Data are shown as means ± SEM

Since we observed that pharmacological activation of autophagy could help clear away ATXN7Q65, we next investigated whether expression of mutant ATXN7 resulted in increased endogenous autophagic activity. However, analysis of the autophagy marker LC3 II (Mizushima et al. 2010; Rubinsztein et al. 2009) in FLQ65 PC12 cells induced to express ATXN7Q65-GFP for different time lengths revealed no significant increase in the level of LC3 II suggesting that the PC12 cells have not up-regulated autophagy despite expressing ATXN7Q65-GFP (Fig. 7).

### Pharmacological Induction of Autophagy Ameliorates ATXN7-Induced Toxicity

**Fig. 8:**
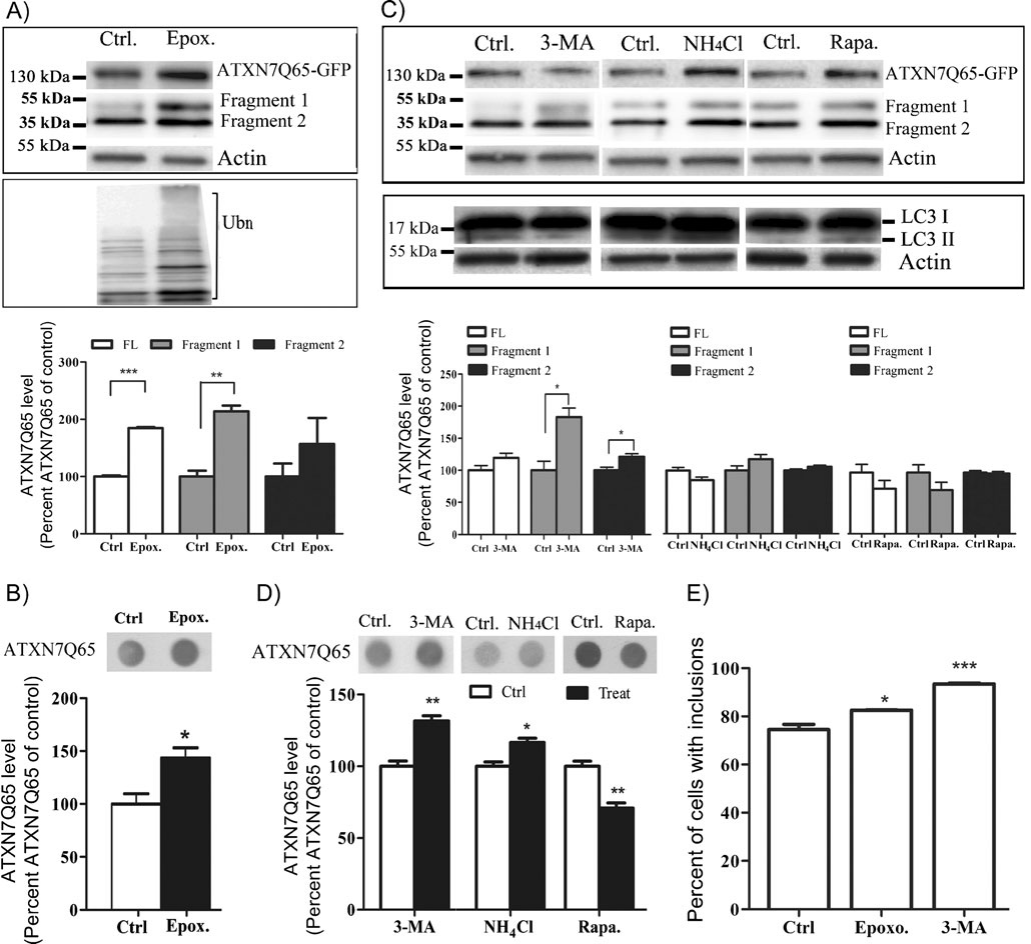
UPS and autophagy both contribute to the clearance of ATXN7 species in PC12 cells continuously expressing ATXN7Q65-GFP. The expression of ATXN7Q65-GFP was induced in FLQ65 PC12 cells for 8 days and the cells treated with UPS or autophagy inhibitors or activators for the last 24–48 h. Induced but non-treated cells were used as control. **a** Epoxomycin treatment for 24 h. *Top panel*: representative ATXN7 blot, *middle panel*: probing for poly-ubiquitinated proteins to verify UPS inhibition, and *bottom panel*: quantification of ATXN7. **b** Analysis of aggregated ATXN7 material after 24 h of UPS inhibition. *Top panel*: representative blot, *bottom panel*: quantification. **c** Inhibition of autophagy with 3-MA for 24 h or NH_4_Cl for 48 h, as well as activation of autophagy with rapamycin for 48 h. *Top panel*: representative ATXN7 western blots, *middle panel*: probings for LC3 to verify the effect of the treatment on autophagy, and *bottom panel*: quantification of ATXN7. **d** Analysis of aggregated ATXN7 material after the same treatments as in **c**. *Top panel*: representative ATXN7 blot, *lower panel* quantification. **e** The number of cells with ATXN7-positive inclusions after UPS and autophagy inhibition. Non-treated cells were used as control. All quantifications were done from three independent experiments, and for analysis of soluble ATXN7 levels, the ATXN7 intensity was normalized against actin (loading control). Data are shown as means ± SEM. *p < 0.05; **p < 0.01; ***p < 0.001

**Fig. 9:**
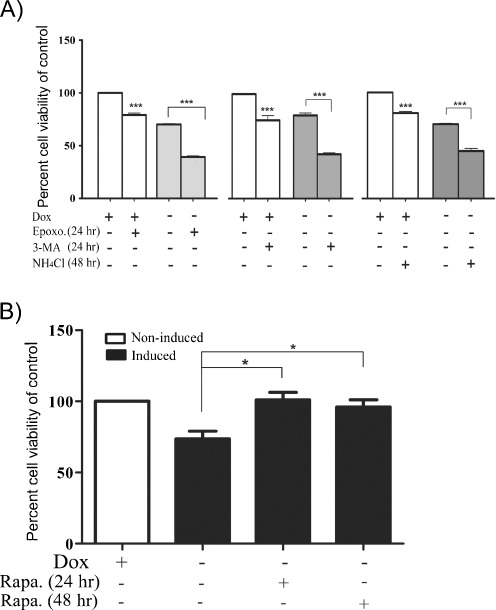
Pharmacological activation of autophagy ameliorates the ATXN7Q65 toxicity. The viability of non-induced or FLQ65 cells induced to express ATXN7Q65-GFP for 8 days while treated with inhibitors or activators for the last 24–48 h as indicated in the figure was analyzed. As controls, non-induced or induced non-treated cells were used. The cell viability was analyzed using the WST-1 assay and normalized against the protein concentration. **a** The induction of ATXN7Q65-GFP expression caused an approximately 20% decrease in cell viability compared to the non-induced control. Treatment of ATXN7Q65-GFP expressing cells with the UPS inhibitor epoxomycin or the autophagy inhibitors 3-MA and NH_4_Cl leads to a further circa 30.1%, 37.0%, and 25.0% decrease in cell viability. Decreases in cell viability were also seen after treatment of non-induced FLQ65 cells; however, the effect of inhibitors was statistically greater in ATXN7Q65-GFP expressing cells. **b** Treatment of ATXN7Q65-GFP expressing cells with the autophagy activator rapamycin for 24 or 48 h restored viability of the ATXN7Q65-GFP cells to the same level as in non-expressing ATXN7Q65-GFP cells. All quantifications were done with data from three independent experiments and data are shown as means ± SEM. *p < 0.05; ***p < 0.001

To further investigate the role of UPS and autophagy in ATXN7 turnover and toxicity, the levels of soluble monomeric and aggregated ATXN7, as well as cell viability, were compared in FLQ65 PC12 cells allowed to continuously express ATXN7Q65-GFP for 8 days while treated with or without UPS or autophagy inhibitors/activators for the last 24 or 48 h (Fig. 8). Consistent with previous results, only inhibition of UPS with epoxomycin elevated the levels of full-length monomeric ATXN7Q65-GFP when ATXN7 was continuously expressed in PC12 cells (Fig. 8a). An increase in the level of the Q65 fragment 1, as well as an increase in aggregated ATXN7 material, was also observed (Fig. 8a, b). Microscopic analysis further confirmed the increase in aggregated ATXN7 material as a 7.65 ± 5.12% increase in the number of cells with inclusions was observed after UPS inhibition (Fig. 8e). Analysis of cell viability revealed that UPS inhibition decreased the viability of ATXN7Q65-GFP cells by 30.1% (Fig. 9a). UPS inhibition also decreased the viability of non-induced FLQ65 cells. However, the effect on viability was statistically larger in ATXN7Q65-GFP expressing cells. These data suggest that clearance of ATXN7Q65-GFP by UPS reduces ATXN7-induced toxicity.

Similarly to what was observed during the clearance experiments, neither activation nor inhibition of autophagy had any effect on monomeric full-length ATXN7Q65-GFP levels (Fig. 8c). However, inhibition of autophagy by 3-MA resulted in elevated levels of both Q65 fragments (Fig. 8c) as well as increased the level of aggregated ATXN7 material and the number of cells with inclusions by 16.28 ± 3.83% (Fig. 8d, e). An increase in aggregated ATXN7 material was also observed after NH_4_Cl treatment (Fig. 8d). Inhibition of autophagy by 3-MA or NH_4_Cl reduced the viability of ATXN7Q65-GFP expressing cells with 37% and 25%, respectively (Fig. 9a). Autophagy inhibition also affected the viability of non-induced cells, but the effect was statistically larger in ATXN7Q65-GFP expressing cells than in non-induced FLQ65 cells. In contrast, activation of autophagy with rapamycin decreased the level of aggregation and restored the viability in ATXN7Q65-GFP expressing cells to the same level as in non-induced PC12 cells (Figs. 8d and 9b). Together these data suggest that clearance via both UPS and autophagy is important to decrease the toxicity of mutant ATXN7, and up-regulation of ATXN7 degradation could be used to ameliorate the toxicity.

## Discussion

Expanded polyglutamine proteins have been suggested to be resistant to UPS degradation and cause toxicity through a gain-of-function mechanism (Bence et al. 2001; Holmberg et al. 2004; Jana et al. 2001). Pharmacological activation of autophagy has been shown to increase the degradation and ameliorate the toxicity of some but not all polyglutamine proteins (Menzies et al. 2010; Nisoli et al. 2010; Ravikumar et al. 2004; Tanaka et al. 2004). To date, few reports investigating the degradation pathways of ATXN7 and the effect of autophagy activation on SCA7 toxicity have been published. In this study, we applied UPS and autophagy inhibitors/activators to determine the role of UPS and autophagy in the degradation and toxicity of the SCA7 disease protein ATXN7. We show that endogenous and transgenic ATXN7 carrying a normal Q10 repeat are primarily degraded by the UPS in both HEK 293T and PC12 cells. UPS also had a significant effect on the clearance of mutant ATXN7Q65 in both cell types. However, a clear contribution by autophagy in the clearance of cleavage fragments of the mutant protein and aggregated ATXN7 material was also observed. Furthermore, pharmacological activation of autophagy by rapamycin ameliorated mutant ATXN7Q65-GFP-induced toxicity in a novel stable inducible PC12 cell line.

Proteolytic processing resulting in smaller more toxic fragments carrying the polyQ domain has been suggested to contribute to the pathology in several polyQ diseases. Proteolytic cleavage products of ATXN7 have been found in SCA7 patients as well as in transgenic models, and caspase-7 cleavage of ATXN7 at amino acids 266 and 344 has been identified (Garden et al. 2002; Young et al. 2007; Yvert et al. 2001). In our study, we could observe N-terminal ATXN7 fragments carrying the polyQ domain in western blot extracts from both transfected HEK 293T cells and stable inducible PC12 cells. One predominant fragment was seen in our ATXN7Q10 cells, whereas two main fragments of approximately 40 and 45 kDa were observed in ATXN7Q65 cells. Taking advantage of the ability to switch the ATXN7 expression off in our novel stable PC12 cell models, we compared the clearance rate and degradation pathways of full-length and ATXN7 fragments. We found that full-length GFP-tagged ATXN7Q10 and the Q10 fragment were degraded by UPS and had estimated half-lives of 4.5 and 4.7 h. These estimated half-lives are consistent with previous studies indicating a short half-life for wild-type ATXN7 (Yoo et al. 2003; Yvert et al. 2001). The preferential degradation of wild-type ataxin-7 by UPS was also confirmed in HEK 293T cells.

Soluble forms of both full-length and fragments of mutant ATXN7 appeared to be more stable than the respective wild-type forms, as no clearance but rather an increase was initially observed after turn off of additional mutant ATXN7 expression in the PC12 cells. This finding is consistent with a previous study showing accumulation of soluble ATXN7 in an animal model (Yvert et al. 2001). However, after a few hours, we could observe a rapid clearance of both soluble full-length and mutant fragments in our PC12 cells. This clearance is most likely due to aggregation, as we could observe a progressive increase in aggregated ATXN7 material during the same time period. The half-life of aggregated ATXN7 material (full-length and/or fragments) was estimated to be approximately 34 h showing that aggregation is a major factor in stabilizing mutant ATXN7 against degradation.

Interestingly, UPS and autophagy inhibition had different effects on the various species of mutant ATXN7. While UPS inhibition increased the levels of soluble full-length and ATXN7 fragments as well as aggregated ATXN7 material, autophagy inhibition only affected the levels of ATXN7 fragments and aggregated material. This difference was observed in both the stable PC12 and the HEK 293T cells. These data suggest that UPS is the main pathway for degradation of full-length mutant ATXN7, while both UPS and autophagy contribute to clearance of ATXN7 fragments. This is perhaps not surprising, as we could show with cell fractionations and microscopy that full-length ATXN7 is predominantly localized to the nucleus which is devoid of autophagy. In contrast, ATXN7 fragments were shown to localize to the cytoplasm as well as the nucleus and thereby be accessible to the autophagic machinery. Our finding that ATXN7 fragments can be degraded by both UPS and autophagy is in contrast to the findings of Mookerjee et al. who found that neither UPS nor autophagy inhibition had any effect on a mutant ATXN7 fragment transiently expressed in HEK 293T cells (Mookerjee et al. 2009). This discrepancy could be due to the processing of full-length ATXN7 in our cells resulting in different fragments from the one expressed in their study or their longer 92Q repeat domain. The proteolytic cleavage of ATXN7 has been suggested to occur in the nucleus and result in the entrapment of the fragments in the nucleus as they are devoid of any nuclear export signal (Young et al. 2007). Nuclear entrapment would prevent autophagic degradation of ATXN7 fragments. However, this does not seem to be the case in our PC12 cell model, possibly since we have a relatively short polyQ expansion and the fragments produced are small enough to diffuse through the nuclear pore complexes.

Despite the different effects UPS and autophagy inhibition had on the level of soluble full-length mutant ATXN7, inhibition of both systems resulted in a similar worsening of ATXN7-induced toxicity. As both treatments caused elevation in Q65 fragments and aggregated ATXN7 material, this suggests that these species are more toxic than the soluble full-length protein. This is in agreement with Young et al. who showed that expression of truncated ATXN7 fragments caused more aggregation and higher toxicity than expression of full-length ATXN7 in HEK 293T cells (Young et al. 2007). This idea is further supported by our finding that autophagy activation by rapamycin, an mTOR-dependent activator of autophagy (Sarkar et al. 2009), could decrease the levels of aggregated ATXN7 material and ameliorate ATXN7-induced toxicity in our PC12 cell model. This was done without a change in the expression level of soluble full-length ATXN7 indicating that the effect of rapamycin was through the activation of autophagy and not by decreasing ATXN7 protein synthesis as have been reported to occur with the huntingtin protein in an HD model (King et al. 2008). An increase in LC3-positive puncta indicating an induction of autophagy activity has been reported in SCA7 transgenic mice (Mookerjee et al. 2009). However, we could not see any signs of increased endogenous autophagy activity after induction of mutant ATXN7 expression in our stable PC12 cells (Fig. 7). Why the cells do not up-regulate the autophagic activity to protect against the ATXN7 toxicity in our model is unclear. Failure to activate autophagy has been reported in several other neurodegenerative disorders (Wong and Cuervo 2010a), but the mechanism behind this is still unclear.

In conclusion, our study shows that both UPS and autophagy are important for the clearance of mutant ATXN7 and thereby reduce ATXN7-induced toxicity. While clearance of full-length mutant ATXN7 is primarily performed by UPS, autophagy contributes to the clearance of mutant ATXN7 fragments and possibly ATXN7 aggregates. Furthermore, pharmacological activation of autophagy ameliorated the ATXN7 toxicity in our model and might be used as a therapeutic strategy in SCA7.

## Electronic supplementary material

Below is the link to the electronic supplementary material.

Supplementary Fig. S1 The filter retardation assay traps aggregated mutant ATXN7 in the insoluble fractions from cells, but does not detect soluble mutant ATXN7 in soluble fractions. HEK 293T cells transfected with ATXN7Q10-Myc or ATXN7Q65-Myc were harvested using RIPA buffer 48 h after transfection and soluble and insoluble fractions were separated as described in the “Materials and Methods”. **A** SDS-PAGE and western blot of soluble fractions show that ATXN7Q10-Myc and ATXN7Q65-Myc are highly expressed. **B** Quantification of ATXN7 expression from three independent experiments reveals no statistical difference in the expression level of ATXN7Q10-Myc and ATXN7Q65-Myc in the soluble fraction. **C** Filter retardation assay of soluble and insoluble extracts from ATXN7Q10-Myc and ATXN7Q65-Myc transfected cells. No mutant ATXN7 was trapped on the membrane from the soluble fraction (PDF 50 kb)

## Abbreviations

ATXN7: Ataxin-7
GFP: Green fluorescence protein
HD: Hungtinton’s disease
kDa: Kilodalton
PolyQ: Polyglutamine
Q: Glutamine
SCA7: Spinocerebellar ataxia type 7
STAGA: SPT3-TAF(II)31-GCN5L acetylase
UPS: Ubiquitin–proteasome system
