# Supplementary material for: Differential Degradation of Full-length and Cleaved Ataxin-7 Fragments in a Novel Stable Inducible SCA7 Model
Source: J Mol Neurosci. 2012 Feb 25;47(2):219–33. doi: 10.1007/s12031-012-9722-8 (PMC3360856; doi:10.1007/s12031-012-9722-8)
Supplement: Supplementary file 1 — The filter retardation assay traps aggregated mutant ATXN7 in the insoluble fractions from cells, but does not detect soluble mutant ATXN7 in soluble fractions. HEK 293T cells transfected with ATXN7Q10-Myc or ATXN7Q65-Myc were harvested using RIPA buffer 48 h after transfection and soluble and insoluble fractions were separated as described in the “Materials and Methods”. A SDS-PAGE and western blot of soluble fractions show that ATXN7Q10-Myc and ATXN7Q65-Myc are highly expressed. B Quantification of ATXN7 expression from three independent experiments reveals no statistical difference in the expression level of ATXN7Q10-Myc and ATXN7Q65-Myc in the soluble fraction. C Filter retardation assay of soluble and insoluble extracts from ATXN7Q10-Myc and ATXN7Q65-Myc transfected cells. No mutant ATXN7 was trapped on the membrane from the soluble fraction (PDF 50 kb) [file 12031_2012_9722_MOESM1_ESM.pdf]

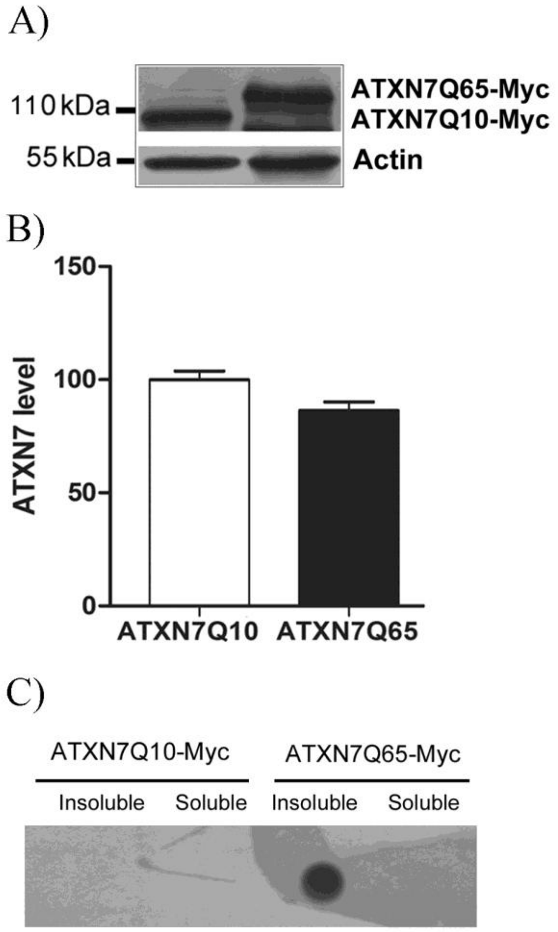

Supplementary Figure S1

The filter retardation assay traps aggregated mutant ATXN7 in the insoluble fractions from cells, but does not detect soluble mutant ATXN7 in soluble fractions. HEK 293T cells transfected with ATXN7Q10-Myc or ATXN7Q65-Myc were harvested using RIPA buffer 48 hours after transfection and soluble and insoluble fractions were separated as described in material and methods. A) SDS-PAGE and western blot of soluble fractions shows that ATXN7Q10-Myc and ATXN7Q65-Myc are highly expressed. B) Quantification of ATXN7 expression from three independent experiments reveals no statistical difference in the expression level of ATXN7Q10-Myc and ATXN7Q65-Myc in the soluble fraction. C) Filter retardation assay of soluble and insoluble extracts from ATXN7Q10-Myc and ATXN7Q65-Myc transfected cells. No mutant ATXN7 was trapped on the membrane from the soluble fraction.
